# Shoulder and Upper Back Pain: An Unusual Initial Presentation of Rare Stage IV Small Cell Neuroendocrine Cervical Cancer

**DOI:** 10.7759/cureus.22708

**Published:** 2022-02-28

**Authors:** Vincent Wong, Monica Abreu-Diaz, Maninderpal Kaur, Sherif El-Hosseiny

**Affiliations:** 1 Internal Medicine - Pediatrics, Rutgers University, New Jersey Medical School, Newark, USA; 2 Internal Medicine, Rutgers University, New Jersey Medical School, Newark, USA

**Keywords:** persistent back pain, neuroendocrine cervical cancer, chronic back pain, metastatic cervical cancer, musculoskeletal manifestations

## Abstract

While musculoskeletal pain is cited as the primary cause of disability and reason for visiting the emergency department in the United States, secondary etiologies should be considered. In this case report, we are reporting a unique case of a 38-year-old multiparous healthy female who presented to multiple emergency departments with fleeting pain on the shoulders and upper back. She was diagnosed with muscle spasms and joint arthritis and discharged home multiple times. The patient then developed vaginal bleeding, belt-line numbness, and was found to have T6 spinal cord compression. Imaging prompted workup for malignancy, which revealed small cell neuroendocrine cervical cancer (SCNECC) with metastasis to intra-abdominal lymph nodes, bone, and brain. SCNECC is very rare, aggressive, occurs in less than 3% of cervical cancers, and does not have established treatment guidelines. Because it is commonly misdiagnosed and has an overall poor prognosis, SCNECC can be missed if it is not part of the differential.

## Introduction

Musculoskeletal pain, especially back pain, is one of the leading causes of visits to physicians [1,2]. Patients have decreased quality of life due to persistent symptoms and the recurring nature of the pain [2]. Too often, patients are sent home with conservative treatments, consisting of over-the-counter analgesics, exercise therapy, and psychosocial interventions without adequate workup [3]. A thorough history and physical examinations are necessary to determine the etiology of the pain, and to screen for “red flags.” These include weight loss, pain that is worse at night, bladder or bowel dysfunction, saddle anesthesia, and pain radiating to the upper limbs, all of which should prompt further investigation [2]. While 90% of back pain is non-specific, some cases are due to more alarming etiologies, such as metastatic cancer, which occurs in 0.7%, infection in 0.01%, and cauda equina syndrome in 0.04% of patients [3,4]. In this case report, we have a patient with multiple episodes of recurrent musculoskeletal pain that did not improve, then developed belt-line numbness and was found to have metastatic small cell neuroendocrine cervical cancer (SCNECC).

## Case presentation

A 38-year-old multiparous female with no past medical history presented to the emergency department with six days of intermittent achy, cramping mid-back pain. Her symptoms started three months prior as bilateral shoulder pain, which went away on its own. Several weeks later, the patient had episodes of upper back pain, which was diagnosed in multiple emergency department visits as muscle spasms and arthritis. One week prior to her hospital admission, the patient’s mid-back pain returned associated with bilateral leg weakness, causing her to fall several times. She denied trauma to her back or legs, numbness or tingling, or urinary or bowel incontinence. The patient, however, did complain of a one-month history of vaginal bleeding varying from blood clots to light spotting without postcoital bleeding, which she attributed to her period.

The patient had six pregnancies with the last delivery over 14 years ago, shortly after which she had a copper intrauterine device placed, which was never removed. She had not seen a primary care doctor or a gynecologist in the last several years but was told she had fibroids. Her periods were regular, and her last menstrual period was about 1.5 weeks prior to presentation. The patient did not recall her last pap smear or had any other sexually transmitted diseases but was treated for an episode of chlamydia in the past. She smoked marijuana frequently but denied using other forms of illicit drugs. Since her initial emergency department visit, she was occasionally taking ibuprofen and cyclobenzaprine as needed for her pain. The patient had a family history of ovarian cancer in her maternal aunt.

In the emergency department, her vitals were stable, and her cardiovascular and respiratory exams were normal. Her strength on the right hip, knee, and foot plantar flexion was 4/5 and was 5/5 on the right knee extension and foot dorsiflexion. Her strength in all other extremities was 5/5. She was moving all extremities spontaneously without any apparent focal deficits. The straight leg raise test was not done. Her neurological exam showed decreased sensation to light touch at the umbilicus and below to the perineal area. No pronator drift was noted and her rectal tone was preserved. A manual pelvic exam was also done but due to body habitus, pain, and positioning, it was able to visualize only the lower half of the cervix, which appeared normal without signs of a mass, bleeding, or discharge. Pap smear was negative for human papillomavirus (HPV) genotypes 16, 18, 31, and 33.

**Figure 1:**
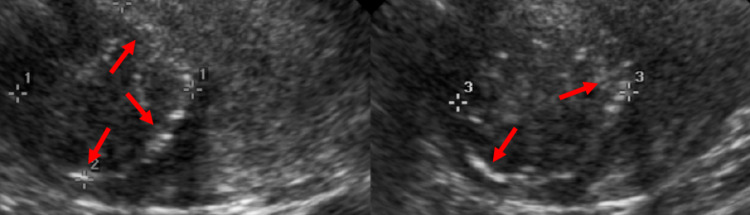
Pelvic ultrasound of the patient’s irregularly shaped uterus, showing a calcified fibroid (red arrows) measuring 5.5 x 5.6 x 5.4 cm.

**Figure 2:**
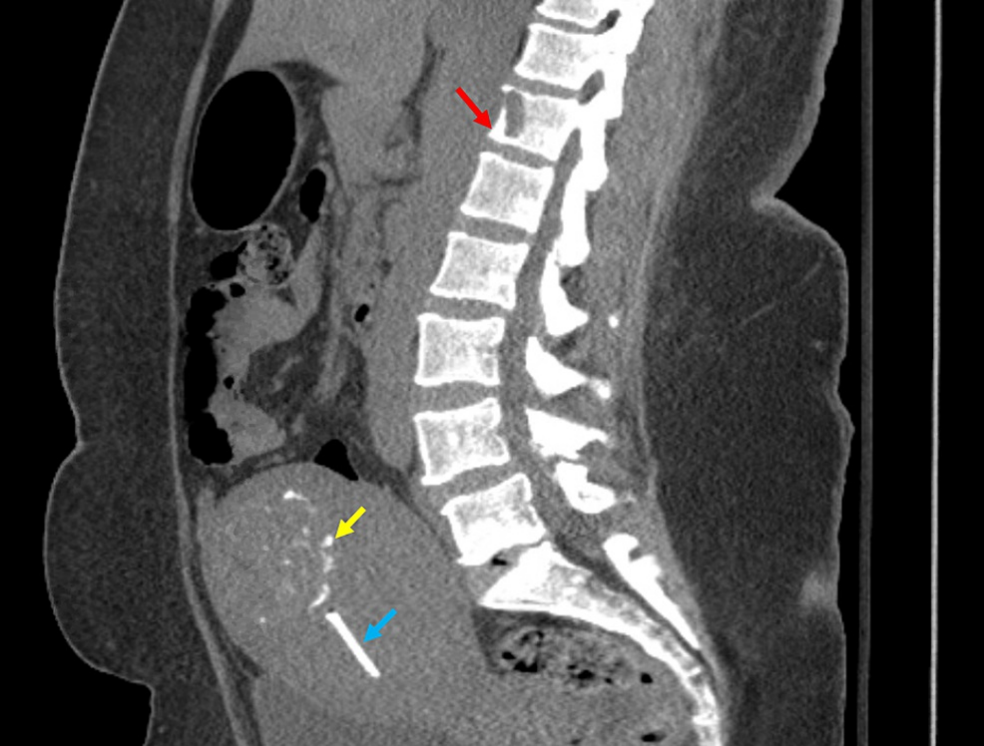
CT scan showing T6 osteolytic lesion (red arrow), calcified fibroid on the uterus (yellow arrow), and intrauterine device in place (blue arrow).

**Figure 3:**
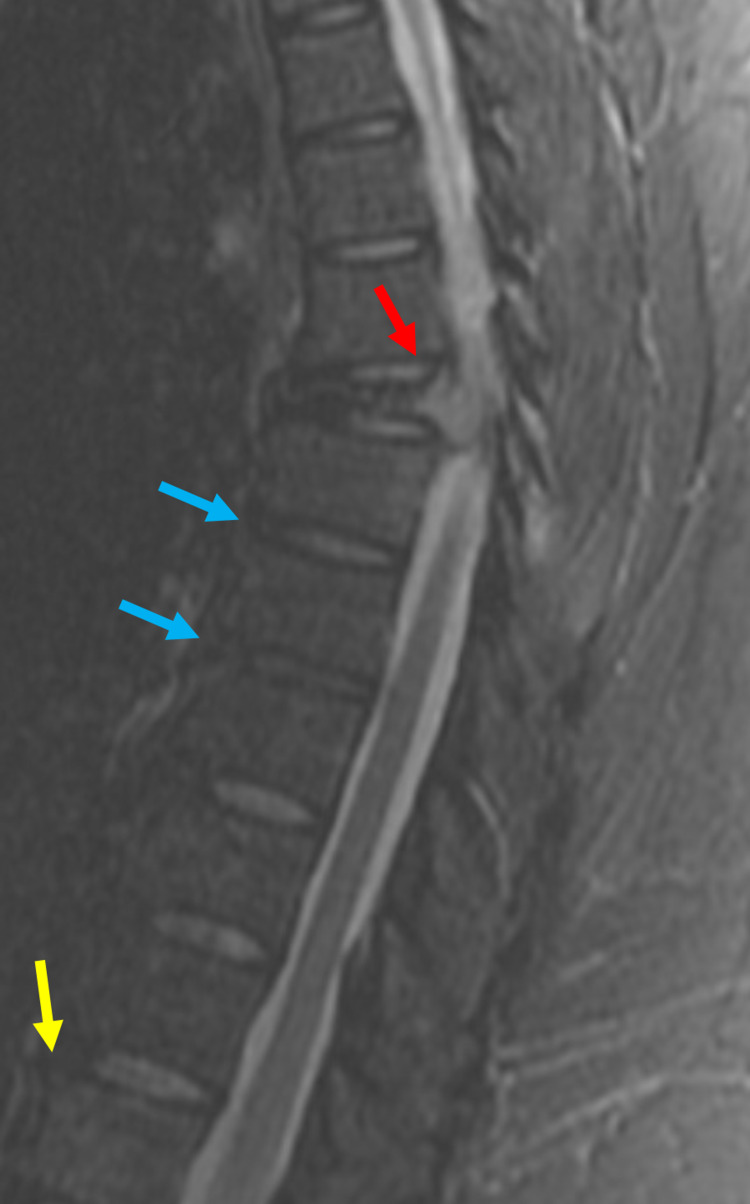
MRI showing loss of height at T6 vertebrae and cord compression (red arrow), demineralization (yellow arrow) at the T12 vertebrae, and diffuse mild spondylosis of the thoracic spine (blue arrows).

**Figure 4:**
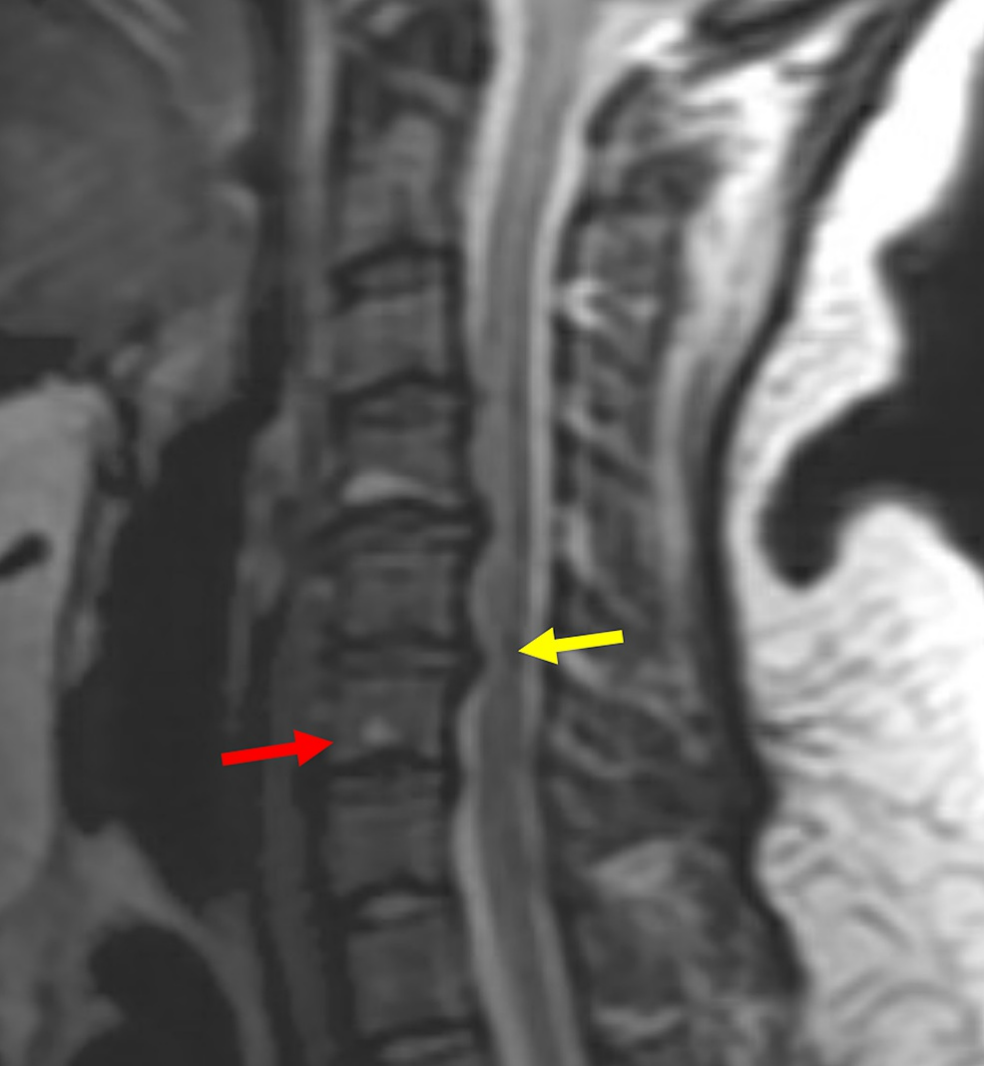
MRI showing cervical spondylosis at C3-C7 abutting the spinal cord (yellow arrow) and enhancing bony lesion at the C6 vertebrae (red arrow).

The patient’s labs were significant for white blood cell count of 16 (normal: 4-11) x10^3/uL, hemoglobin of 11.2 (normal: 12-16) g/dL, and platelet count of 241 (normal: 150-450) x10^3/uL. Sedimentation rate was elevated at 30 (normal: 0-20) mm/hour and C-reactive protein was elevated at 28 (normal: 0-5) mg/L. Prothrombin time (PT), partial thromboplastin time (PTT), and international normalized ratio (INR) were normal. Her chemistry panel and procalcitonin were normal. The serum pregnancy test was negative. The pelvic ultrasound showed a large uterus with multiple calcified fibroids but preserved blood flow (Figure 1). CT scans of her chest, abdomen, and pelvis showed an osteolytic process of the T6 vertebrae with burst fracture, multiple enlarged pelvic and para-aortic lymph nodes, and an enlarged fibroid uterus with calcifications (Figure 2). A follow-up MRI showed cervical spondylosis with posterior ridging at the C3-C7 vertebrae abutting the cervical cord, loss of height at the T6 vertebrae with cord compression, and demineralization at the T12 vertebrae (Figures 3, 4).

**Figure 5:**
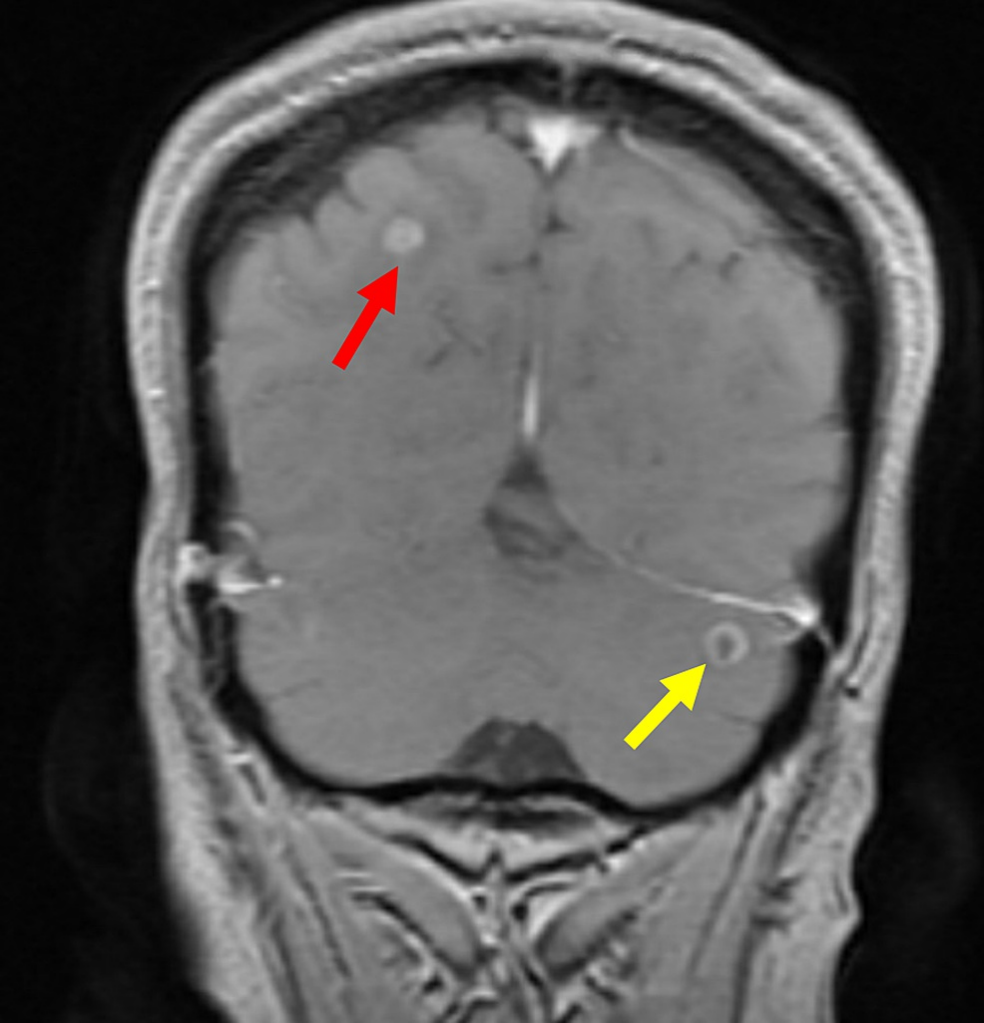
MRI showing metastatic lesions in the cerebrum (red arrow) and cerebellum (yellow arrow).

She was given steroids and taken to the operating room for emergent T5-T7 laminectomy, T4-T8 posterior thoracic fusion, and biopsy of the T6 vertebral lesion. She also underwent a pelvic exam under anesthesia that was significant for a 20+ week-sized globular mobile uterus with an irregular 3-4 cm mass on palpation of the posterior cervix. Brain MRI showed both cerebral and cerebellar metastases (Figure 5). Biopsy of the T6 vertebrae stained strongly positive for synaptophysin, neuron-specific enolase (NSE), chromogranin, and CD56, suggestive of metastatic neuroendocrine cancer. Biopsy of the cervix was positive for NSE, p16, and vimentin, which identified the mass as SCNECC.

The patient was diagnosed with small cell neuroendocrine cancer of the cervix with metastasis to the abdominal lymph nodes, bone, and brain. The neurosurgery, obstetrics-gynecology, gynecological oncology, hematology-oncology, radiation oncology, and internal medicine teams were all involved in her care. She was started on palliative radiation therapy to the thoracic spine and the whole brain and completed 10 sessions in total. Afterward, she was treated with systemic chemotherapy with carboplatin, etoposide, and atezolizumab. The patient was hospitalized for over a month for refractory back pain, subsegmental pulmonary embolism, bacteremia, and neutropenic fever. Giver her poor prognosis, the patient was transferred to hospice care and passed away shortly after.

## Discussion

This patient presented with very common symptoms of shoulder pain that progressed to upper back pain, initially thought to be musculoskeletal in origin. Because she had no other “red flags” and a very low clinical suspicion for malignancy, workup was not started until she had repeated pain that did not resolve with conservative management and neurological symptoms. This prompted imaging, which revealed vertebral bone and disc degeneration in the cervical and thoracic spine, and cord compression due to an underlying malignancy. She was immediately given steroids to decrease inflammation of the spinal cord, subsequent emergent surgical decompression, and further metastatic cancer workup.

The patient was diagnosed with a rare small cell neuroendocrine carcinoma, which makes up less than 3% of all cervical cancers. It can affect women aged 22 to 87 years old, the mean age being 45 years old, with some cases linked to HPV 16 and 18 [5-7]. It often presents as vaginal bleeding, pelvic pressure, or low back pain, with metastasis to regional lymph nodes in the early stages [5,6,8]. Definitive diagnosis is made via biopsy staining for at least two neuroendocrine tumor markers such as synaptophysin, CD56, chromogranin, and NSE [5]. Misdiagnosis on imaging is common for cervical myomas or rapidly growing cervical polyps [6,9]. Treatment is currently limited due to the lack of sufficient research to date [5]. If SCNECC is found in the earlier stages, radical hysterectomy can be performed with neoadjuvant or adjuvant systemic chemotherapy [5]. In the later stages, only radiation and chemotherapy are offered [5]. Chemotherapy regimens are based on those used in small cell lung cancer, consisting of cisplatin and etoposide [5]. With early diagnosis, the prognosis can be up to five years, but as little as one month if diagnosed in the later stages [9,10]. Poor prognosis has also been seen in smokers as well [11].

Unfortunately, the patient was diagnosed with stage IV cancer, metastasizing to the brain and multiple areas of bone including the manubrium, and uniquely to the cervical and thoracic spine. She did not exhibit overt symptoms early on in the course of the disease, which made diagnosis difficult. Her symptoms of vaginal bleeding leading up to presentation could have been masked by the intrauterine device and her uterine fibroids could have confounded the clinical picture. There is a possibility that the fibroids might have been a misdiagnosis but she did not have a further gynecological follow-up.

## Conclusions

The majority of musculoskeletal back pain can be benign, requiring only conservative treatments. However, persistent pain with other systemic symptoms is a "red flag" and requires further workup, especially for malignancy. SCNECC is a rare disease affecting with poor prognosis because of its variable and indolent presentation. Therefore, routine screening and astute clinical suspicion are essential for a timely diagnosis, especially with the high prevalence of HPV and the use of intrauterine devices for contraception.

## References

[1] Weiss AJ, Wier LM, Stocks C, Blanchard J (2006). Overview of emergency department visits in the United States, 2011: statistical brief #174. Healthcare Cost and Utilization Project (HCUP) Statistical Briefs.

[2] Babatunde OO, Jordan JL, Van der Windt DA, Hill JC, Foster NE, Protheroe J (2017). Effective treatment options for musculoskeletal pain in primary care: a systematic overview of current evidence. PLoS One.

[3] Koes BW, van Tulder MW, Thomas S (2006). Diagnosis and treatment of low back pain. BMJ.

[4] Wáng YX, Wu AM, Ruiz Santiago F, Nogueira-Barbosa MH (2018). Informed appropriate imaging for low back pain management: a narrative review. J Orthop Translat.

[5] Tempfer CB, Tischoff I, Dogan A, Hilal Z, Schultheis B, Kern P, Rezniczek GA (2018). Neuroendocrine carcinoma of the cervix: a systematic review of the literature. BMC Cancer.

[6] Pavithra V, Shalini CN, Priya S, Rani U, Rajendiran S, Joseph LD (2014). Small cell neuroendocrine carcinoma of the cervix: a rare entity. J Clin Diagn Res.

[7] Salvo G, Gonzalez Martin A, Gonzales NR, Frumovitz M (2019). Updates and management algorithm for neuroendocrine tumors of the uterine cervix. Int J Gynecol Cancer.

[8] Cohen JG, Kapp DS, Shin JY (2010). Small cell carcinoma of the cervix: treatment and survival outcomes of 188 patients. Am J Obstet Gynecol.

[9] Pan L, Liu R, Sheng X, Chen D (2019). Small cell neuroendocrine carcinoma of the cervix in pregnancy: a case report and review. Case Rep Obstet Gynecol.

[10] Rose PG, Sierk A (2019). Treatment of neuroendocrine carcinoma of the cervix with a PARP inhibitor based on next generation sequencing. Gynecol Oncol Rep.

[11] Chan JK, Loizzi V, Burger RA, Rutgers J, Monk BJ (2003). Prognostic factors in neuroendocrine small cell cervical carcinoma: a multivariate analysis. Cancer.

